# Editorial: Coping With Climate Change: A Genomic Perspective on Thermal Adaptation

**DOI:** 10.3389/fgene.2020.619441

**Published:** 2021-01-13

**Authors:** Margarida Matos, Pedro Simões, Inês Fragata, Ana Sofia Quina, Torsten Nygaard Kristensen, Mauro Santos

**Affiliations:** ^1^cE3c – Centre for Ecology, Evolution and Environmental Changes, Faculdade de Ciências, Universidade de Lisboa, Lisbon, Portugal; ^2^CESAM, Centre for Environmental and Marine Studies, Universidade de Aveiro and Faculdade de Ciências, Universidade de Lisboa, Lisbon, Portugal; ^3^Section of Biology and Environmental Science, Department of Chemistry and Bioscience, Aalborg University, Aalborg, Denmark; ^4^Departament de Genètica i de Microbiologia, Grup de Genòmica, Bioinformàtica i Biologia Evolutiva (GBBE), Universitat Autònoma de Barcelona, Barcelona, Spain

**Keywords:** candidate genes for thermal adaptation, genomic basis of evolutionary potential, genome-wide association studies, transcriptomics, thermal plasticity

Current human-induced climate warming poses a threat to many organisms (Somero, 2012; Buckley and Huey, 2016; Walsh et al., 2019). Species respond to climate change in different ways, from plasticity, evolutionary adaptation, and dispersal, to extinction (Holt, 1990; Parmesan, 2006). In ectotherms, the upper thermal limits have limited plasticity compared to lower thermal limits (Gunderson and Stillman, 2015). Additionally, physiological tolerance to critically high temperatures (when performance drops to zero) may be genetically constrained (Araújo et al., 2013; Hoffmann et al., 2013). Consequently, behavioral thermoregulation can be an important mechanism in buffering exposure to extreme temperatures (Sunday et al., 2014). Predicting how species may adapt to new thermal conditions requires robust ways of evaluating their underlying evolutionary and plastic potentials. Given that intraspecific differentiation to upper critical thermal limits is commonly observed (e.g., Herrando-Pérez et al., 2019, 2020), selection for tolerance to high temperatures may be occurring, although it is not clear how. This calls for a deeper understanding of the underlying genetic basis of thermal adaptation (Porcelli et al., 2015).

In recent decades there has been a boom in studies that use genome-wide sequencing (Savolainen et al., 2013; Ellegren, 2014). Next-generation sequencing techniques, when applied to experimental thermal evolution have contributed to understanding these genomic responses to changing thermal conditions (e.g., Porcelli et al., 2015; Mallard et al., 2018). The combination of genome-wide screenings with more classical approaches could pave the way for an integrative understanding of how populations cope with climate change. This Research Topic aims to: (1) expand knowledge on the genomic basis of thermal adaptation; (2) assess whether and how genetic and genomic diversity can lead to common or different adaptive routes; and (3) discuss ways to improve the contribution of different studies to community-level knowledge.

Logan and Cox suggest that there is moderate heritability for upper thermal tolerance and, hence, the potential for heat tolerance to evolve. However, this may also be constrained by unfavorable genetic correlations with other thermal performance traits. They also suggest that the plastic response of the transcriptome depends on the magnitude of thermal shifts. Rodrigues and Beldade also study genomics and transcriptomics of plasticity, which are usually assumed to have the potential to enhance thermal adaptation. However, more research is needed because high phenotypic plasticity may be maladaptive in warmer and more variable future climates (Kreyling et al., 2019).

Using a genome-wide association study and transcriptomic profiling in lines from the *Drosophila* Genetic Reference Panel (DGRP, Mackay et al., 2012), Lecheta et al. found that heat tolerance is less variable than cold tolerance, with ~50% more identified genes affecting the latter. These results reinforce that heat tolerance is more constrained than cold tolerance (Araújo et al., 2013). However, all lines came from one single population, which could produce biased estimates of heat tolerance (Herrando-Pérez et al., 2020), and further research in other populations is needed. The Research Topic also includes genome-wide approaches and gene expression analyses. Sørensen et al. studied *D*. *simulans* populations after 20 generations of experimental evolution under predictable or unpredictable thermal fluctuations. The strongest response involved unpredictable fluctuations, and the genes under selection were distinct from the genes that are important for the adaptive plastic response under predictive thermal fluctuations. Other studies have shown that constant and fluctuating temperatures induce different plastic and evolutionary responses (Botero et al., 2015; Dey et al., 2016), and more studies like this are important in uncovering the complexities of thermal evolution.

A potential limitation of studies on the evolution of heat tolerance is that most ignore the possible negative impacts of sublethal temperatures on oogenesis and spermatogenesis. This could lead to a higher vulnerability to climate warming in many organisms than is currently thought (David et al., 2005; Walsh et al., 2019). Using the DGRP, Zwoinska et al. found that males are more affected than females when flies were exposed at high sublethal temperatures. At the same time, they did not find additive genetic variance for reproductive performance at these temperatures. Similar results were obtained for the egg-to-adult viability assessed at different temperatures in *D. melanogaster* (Kristensen et al., 2015). However, in Zwoinska et al. the power to map the genetic variants of relatively small effects may be reduced due to the low line number. Thus more studies, using several populations or more DGRP lines are needed.

Few studies have demonstrated that adaptive evolution is occurring as a consequence of climate change. Latitudinal and long-term trends in the frequency of inversions in *Drosophila subobscura* are remarkably consistent worldwide and highly correlated with environmental temperature, respond to seasonal changes and frequency shifts shortly after a heatwave (Balanyà et al., 2006; Rezende et al., 2010; Rodríguez-Trelles et al., 2013). Karageorgiou et al. focused on the breakpoints of a particular inversion that shows cyclic seasonal changes and speculate that this might be partly due to antagonistic pleiotropic effects on reproduction and immunity resulting from a position effect affecting the expression of functional genes located at the breakpoints.

An important ingredient in a warming world is the ecological and evolutionary implications of parasite-host dynamics and prevalence. Mazzucco et al. focussed on the endosymbiont *Wolbachia* infecting *D. melanogaster* lines evolved in cold and hot environments, and found that these dynamics cannot be straightforwardly linked to temperature, making it difficult to predict the impact of climate change.

A goal of genome-wide analyses is to detect and understand the signatures left by natural selection on the genome. Cortés et al. summarize some of the tools available to reveal the genetic consequences of climate change, but there are some shortcomings to linking fitness relevant genes with environmental factors; e.g., those related to data reporting as highlighted by Waldvogel et al. This is important, as genotype-environment associations can be a key ingredient in forecasting the response of natural populations to climatic variation.

Relevant insights into the genetic basis of thermal evolution can come from studying the adaptations of organisms that live in extreme natural environments. Using a metagenomics approach, Alcorta et al. shed light on the genomic features and taxonomy of thermophilic cyanobacteria living in hot springs. These features included genome reduction, changes in GC content, coding density, and size of biosynthetic gene clusters.

The contributions to this Research Topic add to our understanding of thermal adaptation and its multifactorial nature, and highlight the challenges that are still ahead of us in striving for a deeper understanding of adaptation to expected higher and more variable future temperatures. As shown in this Research Topic, increased knowledge should be brought about by complementary approaches comprising different levels of biological organization and their interaction, using a variety of methodologies and study organisms.

## Author Contributions

All authors listed have made a substantial, direct and intellectual contribution to the work, and approved it for publication. All authors contributed to the writing of the editorial.

## Conflict of Interest

The authors declare that the research was conducted in the absence of any commercial or financial relationships that could be construed as a potential conflict of interest.
